# Cap-assisted endoscopic sclerotherapy for internal hemorrhoids: technique protocol and study design for a multi-center randomized controlled trial

**DOI:** 10.1177/2631774520925636

**Published:** 2020-06-05

**Authors:** Xia Wu, Quan Wen, Bota Cui, Yafei Liu, Min Zhong, Yu Yuan, Lihao Wu, Xiaoyin Zhang, Yunlian Hu, Muhan Lv, Qianneng Wu, Suyu He, Yan Jin, Shuxin Tian, Rong Wan, Xin Wang, Long Xu, Jianling Bai, Guangming Huang, Guozhong Ji, Faming Zhang

**Affiliations:** Medical Center for Digestive Diseases, Second Affiliated Hospital of Nanjing Medical University, Nanjing, China; Medical Center for Digestive Diseases, Second Affiliated Hospital of Nanjing Medical University, Nanjing, China; Medical Center for Digestive Diseases, Second Affiliated Hospital of Nanjing Medical University, Nanjing, China; Medical Center for Digestive Diseases, Second Affiliated Hospital of Nanjing Medical University, Nanjing, China; Medical Center for Digestive Diseases, Second Affiliated Hospital of Nanjing Medical University, Nanjing, China; Department of Gastroenterology, First Affiliated Hospital of Guangdong Pharmaceutical University, Guangzhou, China; Department of Gastroenterology, First Affiliated Hospital of Guangdong Pharmaceutical University, Guangzhou, China; Department of Holistic Integrative Medicine, Third People’s Hospital of Shenzhen, Shenzhen, China; Department of Gastroenterology, Hubei Provincial Hospital of Traditional Chinese Medicine, Wuhan, China; Department of Gastroenterology, Affiliated Hospital of Southwest Medical University, Luzhou, China; Department of Gastroenterology, Hangzhou Xixi Hospital, Hangzhou, China; Fourth Department of the Digestive Disease Center, Suining Central Hospital, Suining, China; Department of Gastroenterology, Affiliated Hospital of Wuxi No. 2 People’s Hospital of Nanjing Medical University, Wuxi, China; Department of Gastroenterology, First Affiliated Hospital of Shihezi University Medical College, Shihezi, China; Department of Gastroenterology, Shanghai General Hospital, Shanghai Jiao Tong University School of Medicine, Shanghai, China; Department of Gastroenterology, Tangdu Hospital, Fourth Military Medical University, Xi’an, China; Department of Gastroenterology and Hepatology, Shenzhen University General Hospital, Shenzhen, China; Department of Biostatistics, School of Public Health, Nanjing Medical University, Nanjing, China; Medical Center for Digestive Diseases, Second Affiliated Hospital of Nanjing Medical University, Nanjing, China; Key Lab of Holistic Integrative Enterology, Nanjing Medical University, Nanjing, China; Medical Center for Digestive Diseases, Second Affiliated Hospital of Nanjing Medical University, Nanjing, China; Key Lab of Holistic Integrative Enterology, Nanjing Medical University, Nanjing, China; Professor, Medical Center for Digestive Diseases, Second Affiliated Hospital of Nanjing Medical University, 121 Jiangjiayuan, Nanjing 210011, China; Key Lab of Holistic Integrative Enterology, Nanjing Medical University, Nanjing, China

**Keywords:** cap-assisted endoscopic sclerotherapy, endoscopy, hemorrhoids, prolapse, randomized controlled trial, sclerotherapy

## Abstract

**Background:**

Cap-assisted endoscopic sclerotherapy is a new interventional therapy for internal hemorrhoids and rectal prolapse under colonoscopy. The proper length of the endoscopic injection needle is the core for performing cap-assisted endoscopic sclerotherapy well with more benefits and less complications. However, no data are currently available to guide endoscopists to consider the length of injection needle before cap-assisted endoscopic sclerotherapy. This study is designed to evaluate the efficacy and safety of cap-assisted endoscopic sclerotherapy with long or short injection needle in the treatment of internal hemorrhoids.

**Methods:**

This is a nationwide multi-center, prospective, single-blind and randomized controlled trial. Patients with grade I-II internal hemorrhoids who have failed to conservative treatments and grade III internal hemorrhoids who are not suitable for surgery or refuse surgery will be included. Participants will be randomized 1:1 into either long or short injection needle group. The primary outcome is the recurrence rate of internal hemorrhoids 24 weeks after cap-assisted endoscopic sclerotherapy. The secondary outcomes are as follows: (1) symptom severity score, (2) three-level EuroQoL five dimensions health scale scores, (3) occurrence of adverse events and severe adverse events, and (4) patients’ attitudes toward cap-assisted endoscopic sclerotherapy. Data collection will be conducted before and during operation, the 1st day, 1st week, 2nd week, and 24th week after cap-assisted endoscopic sclerotherapy.

**Discussion:**

The outcome of this study is expected to provide a practical clinical protocol of cap-assisted endoscopic sclerotherapy for patients with internal hemorrhoids and promote the use of this new endoscopic technique.

**Trial registration:**

ClinicalTrials.gov, NCT03917056. Registered on 12 April 2019.

## Background

Hemorrhoids are collection of submucosal, fibrovascular, arteriovenous sinusoids that are part of the normal anorectum.^1^ Symptomatic internal hemorrhoids are usually characterized by painless bleeding after defecation, prolapse, anal itching/dampness perianal discomfort and soiling.^1^ However, the reasons why internal hemorrhoids become symptomatic remain controversial. Nowadays, globally recognized that internal hemorrhoids result from the sliding and deterioration of the connective tissue of anal cushions, together with the stagnation of blood inside the dilated hemorrhoidal plexus.^2,3^ Treatment of internal hemorrhoids depends on symptoms and the degree of it, whose substantial progress has been made in the past few decades.^4^ Office treatments or surgical treatments should be considered when conservative treatments such as dietary modification, lifestyle changes, and medical therapies do not respond well.^5^ To maintain the integrity of anal cushion, reduce the postoperative pain, save medical cost, and preserve patients’ working days, patients prefer to choose the office-based procedures like rubber-band ligation (RBL), injection sclerotherapy, infrared coagulation, laser photocoagulation, and others.^1,5,6^ However, clinical trials investigating the effectiveness of treatments for internal hemorrhoids lack uniformity of outcome measurement, which resulted in a wide variety in outcomes between studies and a debate regarding the best treatment option for each grade of internal hemorrhoids.^7^

The RBL and injection sclerotherapy are the mainstay of office treatments.^5,8^ The RBL is cheap and effective, while the high recurrence rate, repeat banding, severe late bleeding, and postoperative pain lower patients’ satisfaction.^9–11^ Injection sclerotherapy represents as a safe and simple palliative treatment for internal hemorrhoids,^1,12,13^ with a relatively low occurrence of post-procedural pain and bleeding.^1,5,14^ However, misplaced injections may result in iatrogenic risks, including pain, perianal abscess, impotence, prostatitis, mucosal ulcer, prostate abscess, rectourethral fistula, and other complications.^1,5,15^ Importantly, as we know, cap-assisted endoscopic sclerotherapy (CAES) has not been used in Europe and North America. The reports on sclerotherapy from those areas should refer to traditional office procedure. Inverted operation with anoscope often used in traditional injection sclerotherapy, which has a blind area, affecting precise operation. Therefore, a novel technique called CAES was designed for internal hemorrhoids in virtue of interventional flexible endoscopy.

The CAES is an innovative endoscopic sclerotherapy procedure that is superior to traditional injection sclerotherapy in the following aspects. First, the cap added to the front of colonoscope can fully expose the operating field. Second, before or during the opportunity of CAES, endoscopist can perform endoscopic differentiation diagnosis (such as tumors, inflammatory bowel disease, and others induced hematochezia) and endoscopic therapy within lower gut based on the same colon preparation, thus saving patients’ medical cost, and physical and mental pain. The last but not least, specially designed length of endoscopic injection needle (e.g. 14–20 mm) that was used in CAES could be helpful for accurately controlling the injection angle, direction, and depth under direct vision, and to avoid iatrogenic injury due to ectopic injection.^12,16^ Our previous studies have shown that CAES is effective in treating internal hemorrhoids, with fewer adverse reactions and higher patient satisfaction,^12,16^ and CAES has been carried out in many hospitals in China. However, the length of marketing available endoscopic needle generally ranges 3 to 5 mm. Due to the shallow injection depth, we hypothesize that the sclerosing agents may cause complications such as artificial ulcer and secondary bleeding. In addition, using short injection needle may need retroflection that would affect precise operation or damage endoscope. In general, the above shortcomings can be overcome by CAES based on long injection needle, which might be conducive to the hemostatic effect and the improvement of the prolapse symptoms of hemorrhoids.

However, there is no solid clinical evidence to support the differences between the use of long or short injection needle during CAES. Therefore, a nationwide multi-center (centers and participants involved in the trial were listed in Supplementary Data), prospective, single-blind, and randomized controlled trial was designed to evaluate the efficacy and safety of CAES with long or short injection needle in the treatment of internal hemorrhoids, to provide reliable evidence for popularization of this minimally invasive technology.

## Methods

**Figure 1.**
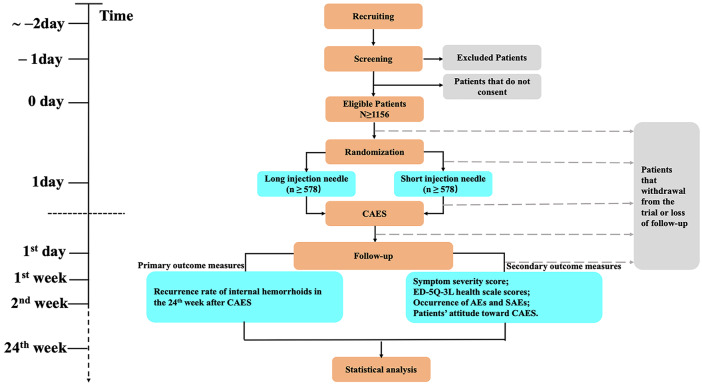
Flowchart of the trail.

**Figure 2.**
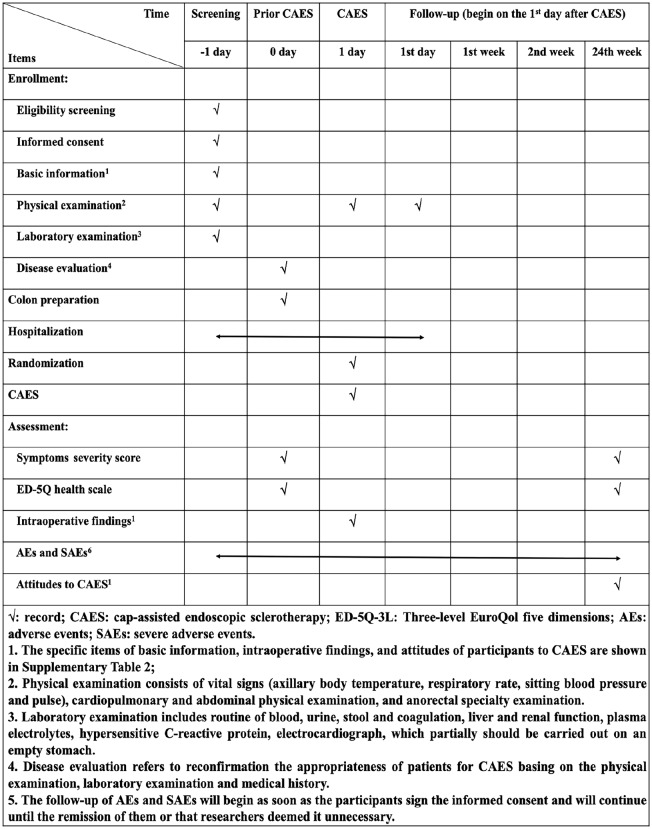
The procedure and checklist of the trial.

This nationwide multi-center, prospective, single-blind, and randomized controlled trial will be conducted in China. Medical Center for Digestive Diseases of the Second Affiliated Hospital of Nanjing Medical University is the responsible unit for this study. Flowchart of the trial is shown in Figure 1. The procedure and checklist of the protocol are displayed in Figure 2.

Informed consent will be obtained from each case. A screening visit will be carried out to ensure the patient eligibility. The participants will undergo colon cleaning prior to CAES and be individually randomized to the long injection needle (14 mm, 23G, FMT-CAES/1800/14, FMT medical, Nanjing, China) or the short injection needle (4 mm, 23G) group. Data collection will be conducted before and during operation, the 1st day, 1st week, 2nd week, and 24th week after CAES. The data will include the recurrence of internal hemorrhoids after CAES, the intraoperative conditions, the incidence of adverse events (AEs), the patients’ attitudes to CAES, and the completion/termination of the trial.

### Inclusion criteria

Patients of any age with grade I-II internal hemorrhoids (with or without external hemorrhoids) that are troublesome to life after conservative treatment will be eligible to attend the trial. Patients with grade III internal hemorrhoids who are not suitable for surgery or reject surgery will also be included. All patients will be required total colonic cleaning and undergone colonoscopy following with CAES.

### Exclusion criteria

Patients who had preexisting medical conditions, including history of anal/endoscopic sclerotherapy, perianal abscess, stricture, fissure, fistula, fecal incontinence, and inflammatory bowel disease, will be excluded. Hypertensive patients with uncontrolled blood pressure and patients with antiplatelet drugs or anticoagulants, acute diarrhea in the last 24 hours, mental disorders, pregnancy, decompensated cirrhosis, cerebrovascular accidents, any blood coagulation dysfunction, and other severe complications (such as severe anal pain with Numerical Rating Scale [NRS] ⩾7) are unsuitable for inclusion. Patients that diagnosed acute thrombotic hemorrhoids or grade IV internal hemorrhoids will be excluded as well.

### Proposed sample size

Sample size calculation was carried out using Stata software system (version 14.0, StataCorp, College Station, TX, USA). Assuming the proportion of patients who experienced recurrence following CAES with long injection needle is 10% and with short injection needle is 16% by reference to previous studies using sclerotherapy for internal hemorrhoids,^12,14,16,17^ the sample size required to detect a difference in the recurrence rates with 80% power and 5% significance is 525 individuals per group. To account for any between-endoscopic operator variation and follow-up loss, we propose increasing this to 578 per group (a 10% increase).

### Classification of internal hemorrhoids

Internal hemorrhoids are graded based on protrusion and reducibility, for which the specific criteria are as follows^5^:

Grade I: prominent hemorrhoidal vessels but no prolapse;
Grade II: hemorrhoids prolapse only with straining and spontaneous reduction;
Grade III: hemorrhoids prolapse beyond the dentate line with straining and require manual reduction;
Grade IV: hemorrhoids prolapse beyond the dentate line with straining and manual reduction ineffective.

### Randomization

A remote, web-based randomization system (Medical Data, Unimed Scientific, Inc, Wuxi, China) will be used to generate random numbers for the participants. Patients will be divided into the long injection needle group (n = 578) and the short injection needle group (n = 578) at a ratio of 1:1 according to the random numbers. The Medical Data application program (APP; System developed by Unimed Scientific, Inc, Wuxi, China) will directly display the randomly assigned treatment group to instruct physicians to select injection needles for CAES.

### Concept and methods of CAES

**Figure 3.**
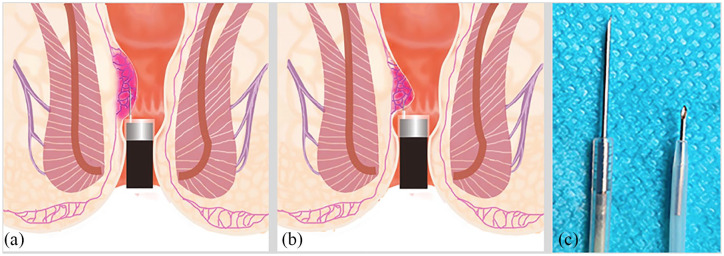
The difference of (a and b) therapeutic procedure, effect, and (c) length between the long injection needle group and the short injection needle group.

**Figure 4.**
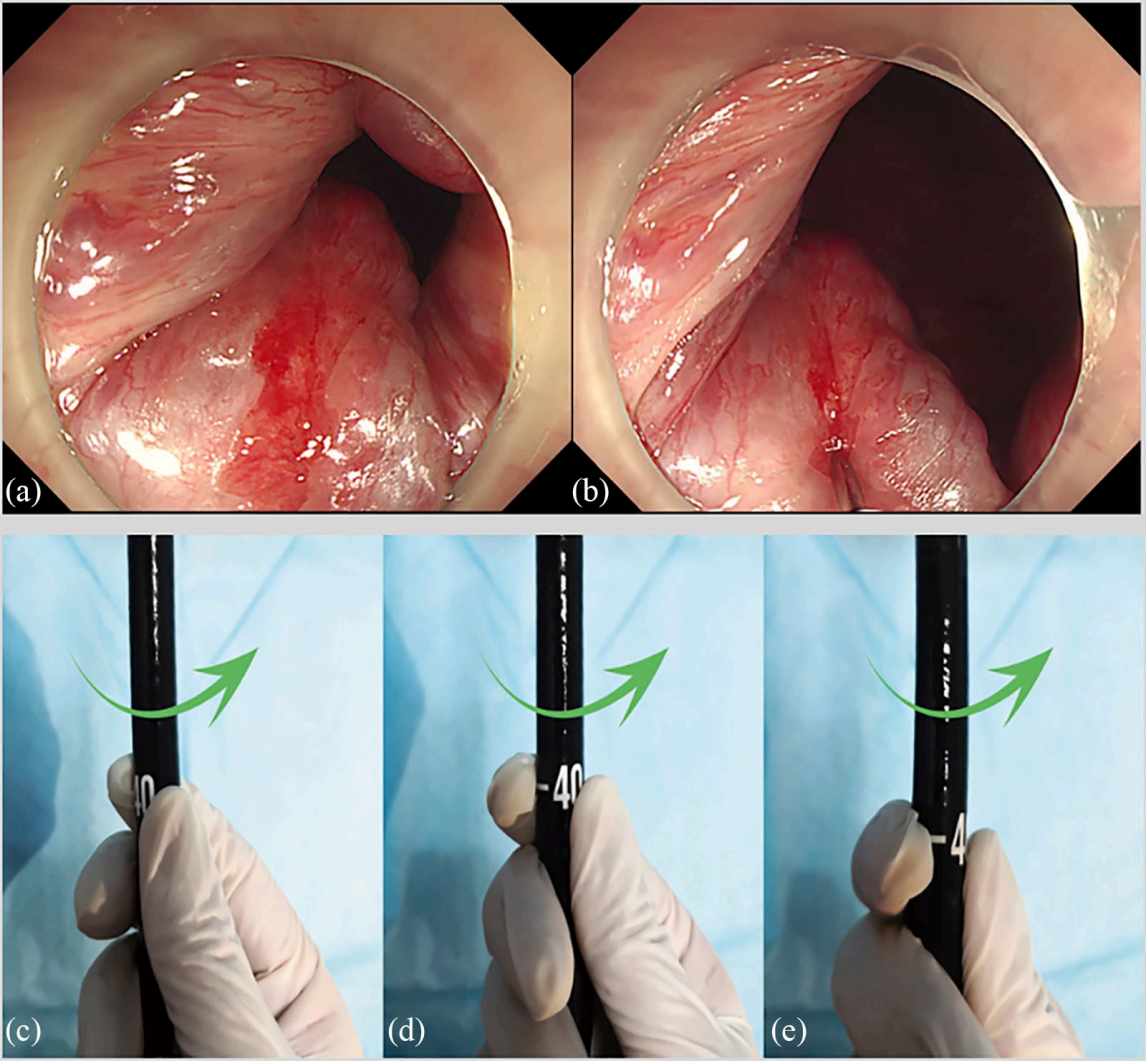
The operation steps of CAES. (a) A full scale of colonoscopy is recommended prior to CAES. (b) The injection needle is advanced to the targeted points through the endoscopic channel to inject the sclerosing agent. (c)–(e) Then choose the injection sites in clockwise order.

Figures 3 and 4 showed the concept of CAES. Table 1 outlined the differences between the CAES based on long injection needle and short injection needle.

**Table 1.** The differences of CAES based on the long and short injection needle.

Items	Long injection needle	Short injection needle
Endoscope	Colonoscope	Colonoscope; gastroscope if retroflection of the endoscope is necessary
Cap	Straight and short	Straight and short
Length of the needle	14 mm	4 mm
Direction of endoscope	Anterograde	Anterograde, retroflection sometimes
Injection position	Above the dentate line	Above the dentate line, the oral side or middle of hemorrhoids
Targeting location	Longitudinal submucosal layer for 14 mm length	Submucosal layer for one point-like bump
Retracting needle	Injecting during retracting the needle	Injecting without retracting the needle
Injection methods	Change injection sites clockwise without tracer, 0.5 to 2.0 mL for each site	Change injection sites clockwise with or without tracer, 0.5 to 2.0 mL for each site
Presumed therapeutic role	Hemostasis and treatment for prolapse	Hemostasis

CAES, cap-assisted endoscopic sclerotherapy.

1. The conventional short and straight transparent cap is fixed on the top of endoscope, which is used for maximizing visibility of the targeting field for diagnosis and injection.
2. A disposable endoscopic injection needle through the endoscopic channel is used to inject the sclerosing agent into submucosal layer.
3. The 6 o’clock position under endoscopic view is the best site for injection. The needle should be advanced to the targeted points, which are above the dentate line to prevent postoperative pain. The sclerosing agent is injected into submucosal layer during 5 seconds when the long needle is slowly removing out of tissue. However, the injection cannot be performed during the withdrawal of short needle. During the procedure, proper air is delivered for the proper exposure of the endoscopic view. Very quick injection and more than 2 mL injection in one site are not permitted, because this seems to increase the risk of huge bump following with artificial ulcer, bleeding, and pain.
4. Before the needle is taken out of injection site, it is required to keep needle stable without moving for at least 5 seconds for preventing bleeding from needle track. No bleeding is the indication of perfect injection.
5. The clockwise order should be followed for choosing the injection sites, then the color tracer is not required to differentiate the injection sites. The methylene blue tracer is not recommended, but it can be used for some beginners under CAES training to differentiate location in case of getting lost in many injection sites.
6. It is required to have enough suction for air and residual fluid in the colon before ending procedure.
7. Both antibiotics and hemostatics generally are not required during the operation period.
8. Patients will be required to stay in the hospital for bed rest on the first night after CAES during this trial for safety control.
9. Medications should be administered in patients complicated with constipation or chronic diarrhea for preventing recurrence of internal hemorrhoids. Suppository drugs are permitted to use when necessary. Medications used should be recorded carefully and taken into consideration when analysis.
10. Patients should be given health education on fiber and fluid intake, bowel patterns (including stool frequency), and bathroom habits (e.g. posture and reading on the toilet).

The sclerosing agent in the present study is Lauromacrogol injection (Tianyu Pharmaceutical, Xi’an, China). Researchers are required to upload the endoscopy report in details after CAES, including the description on procedure, type of needle, and endoscopic images as shown in Supplementary Table 1 to the Medical Data APP. Since 2014 up to 2019, the CAES has been used in more than 500 hospitals for hemorrhoids in China. Researchers involved in the present trial are required to be advanced endoscopists. All researchers must be trained by watching the standard CAES video from Dr Faming Zhang and endoscopy training from advanced CAES experts. At least 5 cases of CAES were performed prior to recruiting case to this study for each researcher.

### Outcome measures

The outcome measures and the follow-up points are shown in detail in the Supplementary Tables. The primary outcome measure is “the recurrence rate,” defined as the proportion of patients with recurrent hemorrhoids at 24 weeks post-CAES, as derived from patients’ self-reported answer to the following questions simplified by the Shanmugam and colleagues’^18^ criteria: “At the moment, do you feel your symptom (anal pain, prolapse, itching, soiling and blood loss) from your hemorrhoids are (1) cured or improved compared with before starting treatment or (2) unchanged or worse compared with before starting treatment.” Patients will be considered to have recurrent hemorrhoids when any of the following are recorded^19^: (1) “Unchanged or worse compared with before starting treatment” at 24th week as reported by the patient, or (2) seeking repeat CAES treatment, alternative non-surgical/surgical treatments for internal hemorrhoids within 24 weeks (except medication treatment), or (3) presence of any symptoms or events that strongly indicated recurrent hemorrhoids among patients not meeting (1) or (2). During follow-up, endoscopic examination will be performed when the patients develop uncontrollable anal pain, bleeding, or when repeat CAES is necessary because of recurrence.

The secondary outcome measures are as follows. (1) Symptom severity score^20^ (Supplementary Table 2): five questions about hemorrhoidal symptoms (anal pain, prolapse, itching, soiling, and blood loss) will be self-assessed by patients by answering how often each symptom was encountered (never, sometimes, weekly, or daily). The score is the sum of the points from all five questions, ranging from 0 to 15 points, where an increase in number is an increase in symptom. Specially, anal pain is described using NRS. Numbers 0 to 10 represent different degree of pain, with Grade 5: 0 = painless, 1~3 = mild pain (sleep is unaffected), 4~6 = moderate pain, 7~9 = severe pain (inability to fall asleep or waking up in sleep), 10 = intense pain. It is worth to note that patients with severe or intense anal pain should be excluded during screening. (2) Three-level EuroQoL five dimensions (ED-5Q) health scale scores: the ED-5Q questionnaire includes five dimensions (mobility, self-care, usual activities, pain/discomfort, anxiety/depression), with three levels in each dimension (no/moderate/severe problem), which has been appeared to be sensitive to changes in patient outcomes in previous studies in this area.^19,21^ Through the Chinese time trade-off (TTO) value table, the health status of five dimensions will be converted into a preference weight of a ED-5Q index score for further analysis.^22^ (3) AEs and SAEs (severe adverse events): AEs refer to adverse medical events that occur during or after CAES, including bleeding, anal pain, having difficulties in passing gas and defecation, urinary retention, infection, ulcer/bleeding in the injection points under endoscopic examination (5–7 days after CAES), and other symptoms. The SAEs include serious complications directly or indirectly related to the CAES, such as death, massive blood loss, incontinence, perforation, fistula, anal stenosis, abscess, and sepsis.^23,24^ (4) Patients’ attitude toward CAES: the survey on the satisfaction with CAES efficacy, the degree of pain relating to CAES, and the willingness to recommend CAES to others in the 24th week.

### Safety assessment and management

The AEs are classified as mild, moderate, and severe according to the degree of impact on daily activity. The relationship between AEs and CAES is divided into five grades, including absolutely relevant, possibly relevant, possibly irrelevant, absolutely irrelevant, and immeasurably, according to the basis of the occurrence time of AEs, symptoms and changes in AEs after cassation/repetition of CAES.

Previous study has shown that CAES using long needle is a safe technique with very few AEs.^12,16^ However, possible risks should be considered for physicians who do not control the angle, direction, and depth of injection under endoscopic view as well. In general, mild AEs caused by CAES can be alleviated by themselves without special intervention. In addition to the following situations: (1) unexplained large amount of bleeding or severe pain after CAES, with ineffective conservative treatments, endoscopic examination should be taken to find etiology and do corresponding treatments; (2) rational use of antibiotics when suspected postoperative infection aggravates; (3) continuous difficulty in passing gas after CAES, enema, blinden, subcutaneous, or intramuscular injection of neostigmine will be helpful; and (4) repeat CAES or other treatment options should be adopted if symptoms are not improved significantly or even worse than before, or the recurrence of internal hemorrhoids.

In case of SAEs, treatments should be given according to the patient’s condition. Timely report to the project sponsor, ethics committee, blinding unit, regulatory authority in accordance with the Sponsor’s Standard Operating Procedures (SOPs). Site staff will be responsible for reporting SAEs and complete an SAE form. The possible most important SAEs is deep infection with pain, swollen, and redness after CAES. Antibiotics should be used in time to control the infection.

### Termination criteria

Completion of the trial or the current data are sufficient to explain the problem to be verified in this trial.

### Statistical analysis plan

Stata software (version 14.0, StataCorp) will be used to analyze the data. The data with the normal distribution will be presented as mean ± standard deviation, while those with abnormal distribution will be displayed as median (interquartile range [IQR]). Differences in the primary outcome measure and recurrence rate of internal hemorrhoids between the two groups will be analyzed by chi-square test. Binary logistic regression, the Wilcoxon rank-sum test, or other tests will be used to analyze the differences and the contributing factors to the possible differences between the two groups. *p* < 0.05 will be considered statistically significant.

### Ethical approval

This trial has been approved by Medical Ethics Committee of the Second Affiliated Hospital of Nanjing Medical University and all ethics committee at other participating centers.

## Discussion

Injection sclerotherapy dates back at least one century and has been regarded as a simple and convenient office procedure for internal hemorrhoids.^25^ However, the development of this minimally invasive therapy has been used much less than before because of ectopic injection. The CAES was coined as an innovation technique for having advantages in accurately controlling the injection angle, direction, and depth under direct vision of flexible endoscope. The core value of CAES for internal hemorrhoids and rectal prolapse is to provide precise therapy, reduce the iatrogenic injuries, and avoid pain during and after therapy. Our pilot studies demonstrated that CAES based on long injection needle is an effective, safe, and convenient operation technique^12,16^; 100% of participants who underwent CAES showed sustained clinical efficacy within the 3-month follow-up, with no severe or obvious complications related to CAES.^16^ The CAES is helpful to avoid doctor’s face close to patient’s anus. Notedly, gastroscope is recommended only for CAES with short injection needle where retroflection of the endoscope is necessary, of which the softer material and thinner diameter make it more flexible in operation. We have to clarify that there is no necessary to perform retroflection when using colonoscope and long needle in pracitce.

The length of injection needle is controversial in the process of injection sclerotherapy. Tomiki and colleagues^15^ suggested that short needle can avoid inserting into dangerous areas such as mucosal muscular layer. They recommend using 3-mm short needle to reduce ectopic injection.^15^ On the contrary, the short injection needle was not suggested in CAES because its short length needs to multiple-site injections, which may lead to more mucosal injury, potential inflammation, and complications such as artificial ulcer and secondary bleeding due to the shallow injection depth and imprecise operation caused by the retroflection of endoscope. Reasonably, the long injection needle would bring more benefit to patients and endoscopists, which requires data to prove its authenticity.

Studies on the treatment of hemorrhoid disease have shown considerable heterogeneity because of the variety of outcome measurements. The outcome measurements we chose based on the Core Outcome Set for hemorrhoid disease developed by Breukink and colleagues and the European Society of Coloproctology may improve the quality of our research and enhance the analysis of evidence.^7^ A previous research indicated that the physical appearance of the postoperative anal cushions and patients’ symptoms are poorly correlated, meaning anorectal visualization is not a reliable surrogate of success.^26^ Our clinical experience and principal management for hemorrhoids shows that there is no need to review colonoscopy within half a year after CAES treatment. We therefore used a simple, dichotomized definition of recurrence based on Shanmugam and colleagues’^18^ systematic review definition and measured this at 24 weeks post-CAES.

Patients might benefit much more from CAES than traditional office injection sclerotherapy. The diagnosis of hemorrhoids is not difficult, but misdiagnosis and missed diagnosis often exist in clinical practice. It is not rare to misdiagnose malignant tumors as hemorrhoids.^1,5,27^ Patients with ulcerative colitis and Crohn’s disease were reported to have hemorrhoids, suggesting that colonoscopy examination before CAES will be conducive to early diagnosis of inflammatory bowel disease with hemorrhoid symptoms by determining intestinal lesions.^28^ In addition, hematochezia can result from hemorrhoids, diverticulosis, mucosal abnormality/colitis, polyp or multiple polyps, tumor, solitary ulcers, and other diseases.^12,29^ Therefore, CAES was originally designed not only for patients who had been clearly diagnosed hemorrhoids that needed therapy but for patients who needed colonoscopy to clarify a diagnosis and then performed possible CAES (patients diagnosed of internal hemorrhoids under this colonoscopy) during the same opportunity of colonoscopy. Moreover, 30.0% and 41.6% of patients underwent other endoscopic procedures such as polypectomy, neoplasia resection, excision of anal papilla fibroma, biopsy during CAES in our previous studies,^12,16^ which undoubtedly reduced patients’ medical costs and the physical and mental pain caused by anesthesia, intestinal preparation

There are some limitations in this study, which were not designed to compare the efficacy and safety of other office-based procedures like RBL. Furthermore, this is a single-blind design study, because the visualization of the operation cannot be double-blind.

## Conclusion

In summary, we assume that long injection needle for CAES could lead to better efficacy and less AEs, and achieve higher patient satisfaction than that observed with short needle. This multi-center randomized controlled study is expected to provide the solid evidence to guide endoscopists to perform CAES for internal hemorrhoids and move CAES forward.

## Supplemental Material

Supplementary_Materials_0329 – Supplemental material for Cap-assisted endoscopic sclerotherapy for internal hemorrhoids: technique protocol and study design for a multi-center randomized controlled trial Click here for additional data file. Supplemental material, Supplementary_Materials_0329 for Cap-assisted endoscopic sclerotherapy for internal hemorrhoids: technique protocol and study design for a multi-center randomized controlled trial by Xia Wu, Quan Wen, Bota Cui, Yafei Liu, Min Zhong, Yu Yuan, Lihao Wu, Xiaoyin Zhang, Yunlian Hu, Muhan Lv, Qianneng Wu, Suyu He, Yan Jin, Shuxin Tian, Rong Wan, Xin Wang, Long Xu, Jianling Bai, Guangming Huang, Guozhong Ji and Faming Zhang in Therapeutic Advances in Gastrointestinal Endoscopy
