# Supplementary material for: Cap-assisted endoscopic sclerotherapy for internal hemorrhoids: technique protocol and study design for a multi-center randomized controlled trial
Source: Ther Adv Gastrointest Endosc. 2020 Jun 5;13:2631774520925636. doi: 10.1177/2631774520925636 (PMC7278306; doi:10.1177/2631774520925636)
Supplement: Supplementary_Materials_0329 – Supplemental material for Cap-assisted endoscopic sclerotherapy for internal hemorrhoids: technique protocol and study design for a multi-center randomized controlled trial [file Supplementary_Materials_0329.pdf]

Supplementary Data China CAES Study Group

1. Bota Cui, Medical Center for Digestive Diseases, the Second Affiliated Hospital of Nanjing Medical University, Nanjing.
2. Jing Cao, Department of Gastroenterology, Changzhi People's Hospital, Changzhi.
3. Jianhui Chen, Department of Gastroenterology, Hubei Provincial Hospital Traditional Chinese Medicine, Wuhan.
4. Jiayu Chen, Department of Gastroenterology, the 940th Hospital of Joint Logistic Support Force of Chinese People's Liberation Army, Lanzhou.
5. Yu Chen, Department of Gastroenterology, Nanhai Hospital of Southern Medical University, Foshan.
6. Mingming Deng, Department of Gastroenterology, the Affiliated Hospital of Southwest Medical University, Luzhou.
7. Zhiqiang Du, Department of Gastroenterology, the Jianyang People's Hospital, Jianyang.
8. Xia Guo, Department of Gastroenterology, Changzhi People's Hospital, Changzhi.
9. Guangming Huang, Medical Center for Digestive Diseases, the Second Affiliated Hospital of Nanjing Medical University.
10. Haibo Hu, Department of Gastroenterology, Changzhi People's Hospital, Changzhi.
11. Suyu He, The Fourth Department of the Digestive Disease Center, Suining Central Hospital, Suining.
12. Yunlian Hu, Department of Gastroenterology, Hubei Provincial Hospital Traditional Chinese Medicine, Wuhan.
13. Guozhong Ji, Medical Center for Digestive Diseases, the Second Affiliated Hospital of Nanjing Medical University, Nanjing.
14. Yan Jin, Department of Gastroenterology, the Affiliated Hospital of Wuxi No.2 People's Hospital of Nanjing Medical University, Wuxi.
15. Min Kang, Department of Gastroenterology, the Affiliated Hospital of Southwest Medical University, Luzhou.
16. Hongguang Li, Department of Gastroenterology, Yiwu Central Hospital, Yiwu.
17. Muhan Lv, Department of Gastroenterology, the Affiliated Hospital of Southwest Medical University, Luzhou.
18. Shancheng Luo, Department of Gastroenterology, the Jianyang People's Hospital, Jianyang.
19. Zheng Liu, Medical Center for Digestive Diseases, the Second Affiliated Hospital of Nanjing

Medical University, Nanjing.

20. Tingfang Ma, Department of Gastroenterology, Changzhi People's Hospital, Changzhi.
21. Wangping Meng, Department of Gastroenterology, the Affiliated Hospital of Southwest Medical University, Luzhou.
22. Hanbing Ning, Department of Gastroenterology, the First Affiliated Hospital of Zhengzhou University, Zhengzhou.
23. Shoubin Ning, Department of Gastroenterology, Air force Medical Center of People's Liberation Army of China, Beijing.
24. Yan Shi, Department of Gastroenterology, Hangzhou Xixi Hospital, Hangzhou.
25. Shuxin Tian, Department of Gastroenterology, the First Affiliated Hospital of the Shihezi University Medical College, Shihezi.
26. Biaomeng Wang, Department of Gastroenterology, the 940th Hospital of Joint Logistic Support Force of Chinese People's Liberation Army, Lanzhou.
27. Jianzhang Wang, Department of Gastroenterology, the Second Affiliated Hospital of Wenzhou Medical University, Wenzhou.
28. Lihao Wu, Department of Gastroenterology, the First Affiliated Hospital of Guangdong Pharmaceutical University, Guangzhou.
29. Qianneng Wu, Department of Gastroenterology, Hangzhou Xixi Hospital, Hangzhou.
30. Rong Wan, Department of Gastroenterology, Shanghai General Hospital, Shanghai Jiao Tong University School of Medicine, Shanghai.
31. Shaofeng Wang, Department of Gastroenterology, Changzhi People's Hospital, Changzhi.
32. Wei Wang, Department of Gastroenterology, the 940th Hospital of Joint Logistic Support Force of Chinese People's Liberation Army, Lanzhou
33. Xin Wang, National Clinical Research Center for Digestive Diseases and Xijing Hospital of Digestive Diseases, Air Force Military Medical University, Xi'an.
34. Xiaohua Wang, Medical Center for Digestive Diseases, the Second Affiliated Hospital of Nanjing Medical University, Nanjing.
35. Jie Xiang, Department of Gastroenterology, the Central Hospital of Enshi Tujia and Miao autonomous Prefecture, Enshi.
36. Long Xu, Department of Gastroenterology and Hepatology, Shenzhen University General Hospital, Shenzhen.
37. Wenrui Xie, Department of Gastroenterology, the First Affiliated Hospital of Guangdong Pharmaceutical University, Guangzhou.

38. Yuan Xiao, Department of Gastroenterology, the Third Affiliated Hospital of Guizhou Medical University, Duyun.
39. Zhang'e Xiong, Department of Gastroenterology, Hubei Provincial Hospital Traditional Chinese Medicine, Wuhan.
40. Caiwen Yan, Department of Gastroenterology, Changzhi People's Hospital, Changzhi.
41. Guodong Yi, Department of Gastroenterology, the Central Hospital of Enshi Tujia and Miao autonomous Prefecture, Enshi.
42. Hong Yin, Department of Gastroenterology, The Fourth People's Hospital of Zigong City, Zigong.
43. Li Yang, Department of Gastroenterology, Jilin Province the First Automobile Works General Hospital, Changchun.
44. Qiong Yan, Department of Gastroenterology, the Affiliated Hospital of Southwest Medical University, Luzhou.
45. Yu Yuan, Department of Gastroenterology, the First Affiliated Hospital of Guangdong Pharmaceutical University, Guangzhou.
46. Faming Zhang, Medical Center for Digestive Diseases, the Second Affiliated Hospital of Nanjing Medical University, Nanjing.
47. Juan Zhang, Department of Gastroenterology, Hubei Provincial Hospital Traditional Chinese Medicine, Wuhan.
48. Shiyang Zhang, Department of Gastroenterology, Mianzhu People's Hospital, Mianzhu.
49. Xiangrong Zhou, Department of Gastroenterology, the Jianyang People's Hospital, Jianyang.
50. Xuesong Zhang, Department of Gastroenterology, Lihuili Hospital of Ningbo Medical Center, Ningbo.
51. Xiaoyin Zhang, Department of Holistic Integrative Medicine, Shenzhen Hospital of Southern Medical University, Shenzhen.

Supplementary Table 1 Case Report Form for CAES

**Time**	Screening	CAES	Follow-up (begin on the 1st day after CAES)			
			1st day	1st week	2nd week	24th week
**Items**						
Basic Information						
Name (acronym)	√	--	--	--	--	--
Gender	√	--	--	--	--	--

Age	√	--	--	--	--	--
Hospital name	√	--	--	--	--	--
Phone number	√	--	--	--	--	--
ID	√	--	--	--	--	--
Address	√	--	--	--	--	--
Height	√	--	--	--	--	--
Weight	√	--	--	--	--	--
History of hemorrhoidectomy	√	--	--	--	--	--
Full name of the hospital	√	--	--	--	--	--
Name of operation physician	--	√	--	--	--	--
Phone number of endoscopist	--	√	--	--	--	--
Treatment Groups (long/short needle)	--	√	--	--	--	--
Symptom score	√	--	--	√	√	√
Anal pain score	√	√	√	√	√	√
AEs and SAEs[1]	√	√	√	√	√	√
ED-5Q-3L scores	√	--	--	--	--	√
CAES record						
Treatment time (Emergency/scheduled)	--	√	--	--	--	--
The final diagnosis (staging)	--	√	--	--	--	--
Polypectomy (Yes/no)	--	√	--	--	--	--
Excision of vegetations (polyps, anal papillary lesions) in the dentate line are (Yes/no)	--	√	--	--	--	--
Excision of pedicled external hemorrhoids or vegetations (Yes/no)	--	√	--	--	--	--
Intraoperative bleeding[2]	--	√	--	--	--	--
Volume of injection	--	√	--	--	--	--
Number of injection sites	--	√	--	--	--	--
Use of medication for chronic diarrhea[3]	--	--	--	√	--	--
Use of medication for chronic constipation[3]	--	--	--	√	--	--
Use of antibiotics[4]	--	--	--	√	--	--
Review of colonoscopy[5]	--	--	--	√	--	--
Recurrence of internal hemorrhoids (Yes/no)	--	--	--	--	--	√

Patients' attitude to CAES						
Satisfaction with CAES efficacy (Very satisfied/General satisfied/Dissatisfied)	--	--	--	--	--	√
The degree of CAES-related pain (Very painful/A little pain/Pain free)	--	--	--	--	--	√
The willingness to recommend CAES to others (Willing/Unwilling)	--	--	--	--	--	√
Seeking repeat CAES, alternative non-surgical/surgical treatments within 24 weeks (except drug conservative treatment)[6]	--	--	--	√	√	√
Completion/termination of the trial[7]	√	√	√	√	√	√

√: record; --: no record; ID: identity card; AEs: adverse events; SAEs: severe adverse events; ED-5Q-3L: Three-level EuroQol five dimensions; CAES: cap-assisted endoscopic sclerotherapy;

1. The time, possible reasons, treatments and outcome of AEs and SAEs should be recorded in detail; e.g., if patients have difficulty in passing gas through anus, the duration and treatment methods (enema, anal tube for decompression, injection of neostigmine) should be recorded in detail.
2. Intraoperative bleeding is evaluated by a 3-grade scale: 1 = no obvious bleeding, 2 = a small amount bleeding that does not affect the endoscopic view, 3 = obvious bleeding that affects the endoscopic view.
3. For patients with chronic diarrhea/constipation, it is recommended to take medication to control/improve symptoms after CAES to prevent bleeding. The name, dosage and course of drugs should be recorded.
4. The use of antibiotics should be considered when suspected postoperative infection aggravates and recorded in detail.
5. On the 5~7 day, whether the participants need colonoscopy reexamination (directly observe rectum after defecation) should be evaluated according to the symptoms. The endoscopic report mainly describes the presence or absence of ulcer/bleeding in the injection points.
6. The reason, time and effect of repeat CAES or other alternative treatments for patients within the 24th week after CAES will be collected.
7. The time and reason to discontinue the trial (select one from a-d) a: participants automatically quit or lose follow-up; b: suspension by researchers; c: unable to continue because of severe adverse events; d: others (please explain the reasons).

Supplementary Table 2 Hemorrhoids symptom score

Frequency<br>Questions	Never*	Sometimes*	Weekly*	Daily*
How often do you experience pain from the hemorrhoids?				
How often do you experience itching or discomfort of the anus?				
How often do you experience bleeding when passing a motion?				
How often do you soil your underwear (mucous, liquid or solid discharge)?				
How often do you have to push back in a prolapse hemorrhoids?				

*Never = no episodes in the past four weeks; sometimes ≥ 1 episodes in the past four weeks but less than once peer week; weekly = 1-6 times per week; daily ≥1 episodes a day.
